# Chronic Unpredictable Stress Decreases Expression of Brain-Derived Neurotrophic Factor (BDNF) in Mouse Ovaries: Relationship to Oocytes Developmental Potential

**DOI:** 10.1371/journal.pone.0052331

**Published:** 2012-12-20

**Authors:** Li-Min Wu, Mei-Hong Hu, Xian-Hong Tong, Hui Han, Ni Shen, Ren-Tao Jin, Wei Wang, Gui-Xiang Zhou, Guo-Ping He, Yu-Sheng Liu

**Affiliations:** 1 Centre for Reproductive Medicine, Anhui Provincial Hospital Affiliated to Anhui Medical University, Hefei, China; 2 Department of Neurology, The First Affiliated Hospital of Anhui College of Traditional Chinese Medicine, Hefei, China; 3 Department of Endocrinology, Anhui Provincial Hospital Affiliated to Anhui Medical University, Hefei, China; VU University Medical Center, The Netherlands

## Abstract

**Background:**

Brain-derived neurotropic factor (BDNF) was originally described in the nervous system but has been shown to be expressed in ovary tissues recently, acting as a paracrine/autocrine regulator required for developments of follicles and oocytes. Although it is generally accepted that chronic stress impairs female reproduction and decreases the expression of BDNF in limbic structures of central nervous system, which contributes to mood disorder. However, it is not known whether chronic stress affects oocytes developments, nor whether it affects expression of BDNF in ovary.

**Methods:**

Mice were randomly assigned into control group, stressed group, BDNF-treated group and BDNF-treated stressed group. The chronic unpredictable mild stress model was used to produce psychosocial stress in mice, and the model was verified by open field test and hypothalamic-pituitary-adrenal (HPA) axis activity. The methods of immunohistochemistry and western blotting were used to detect BDNF protein level and distribution. The number of retrieved oocytes, oocyte maturation, embryo cleavage and the rates of blastocyst formation after parthenogenetic activation were evaluated.

**Results:**

Chronic unpredictable stress decreased the BDNF expression in antral follicles, but didn’t affect the BDNF expression in primordial, primary and secondary follicles. Chronic unpredictable stress also decreased the number of retrieved oocytes and the rate of blastocyst formation, which was rescued by exogenous BDNF treatment.

**Conclusion:**

BDNF in mouse ovaries may be related to the decreased number of retrieved oocytes and impaired oocytes developmental potential induced by chronic unpredictable stress.

## Introduction

Brain-derived neurotropic factor (BDNF) is a member of the nerve growth factor family that is important for neuronal survival and plasticity. BDNF was originally described in the nervous system but has been shown to be expressed in ovary tissues recently [1]–[3]. There are increasing evidences of a role for ovarian BDNF in oocytes development, including oocyte maturation, early embryo cleavage and blastocyst formation [4]–[7].

It is generally accepted that chronic stress impairs female reproduction [8]. Previous studies by our group [9] and others [10] had found that chronic stress leads to follicular maldevelopment [9], [10]. A growing body of evidence demonstrated that chronic stress decreases the expression of BDNF in limbic structures in the central nervous system, which may contribute to mood disorder[11]–[13]. However, it is not known whether chronic stress affects oocytes development, nor whether it affects the expression of BDNF in ovary.

We proposed that ovarian BDNF expression and oocytes development may be affected by chronic stress, and the modulated BDNF is responsible for decreased oocytes development induced by chronic stress. Here, to validate the hypothesis, we used chronic unpredictable mild stress model to produce psychosocial stress in mice. We observed BDNF expression and oocytes development. We further treated these mice with recombinant BDNF, in order to reveal the role of BDNF in the ovarian stress responses.

## Materials and Methods

There are no uses of human or non-human primates in the research. All animal work has been conducted according to relevant national and international guidelines. Animal housing, care, and application of experimental procedure were in accordance with all relevant local guidelines and legislation to minimize pain and suffering of the animals. The research project has got the approval of Ethics Committees on Human Research of Anhui Provincial Hospital, an affiliation to the Anhui Medical University. The permit number was 2008010602.

All chemicals and reagents were purchased from Sigma Chemical Company (St Louis, MO, USA) except for the ones specifically described.

### Animals

A total of 54 5-week-old Swiss female mice were randomly assigned to 4 groups: Control group(n = 18); stressed group(n = 18); BDNF-treated group(n = 9); BDNF-treated stressed group(n = 9). Mice were housed 9 per cage and acclimatized to the animal colony for 1 week before the start of the experimental procedures. The stress group received 30-day stress procedure. All mice received standard rodent diet and tap water ad lib under a 12 h light–dark cycle (lights on 0730–1930) and a constant temperature of 21–22°C and humidity of 55±5%.

### Mouse Stressed Model

Tolerance can develop when rodents are repeatedly exposed to a predictable stressor. However, this does not occur when rodents are exposed to unpredictable stress. A classic stressed model was induced by chronic unpredictable mild stress [9], [14], [15]. The study was conducted in compliance with Ethics Committees on Animal Research of Anhui Provincial Hospital Affiliated to Anhui Medical University. Stressors were administered once daily between 8:30 and 10:30, except the 24 h duration stressors. Stressors consisted of (1) 24 h social isolation (one mouse per cage); (2) 24 h social crowding (18 mice per cage, 325×210×185 mm) plus cage tilt (cages were tilted to 30°C from the horizontal); (3) 1 h warm swim at 31°C; (4) 4 min cold swim at 8–10°C, after which they were toweled dry; (5) 5 min hot stress in oven at 42°C; (6) 24 h food deprivation; (7) 24 h water deprivation with empty drinking bottles; (8) 24 h wet cages; (9) 1 h shaker stress (160 r.p.m.); (10) 24 h light-dark shift. The different stressors were distributed randomly at an interval of 10 days. Every stressor was administered three times within 30 days.

### Treatments and Tissue Preparation

An overdose of BDNF(1 µg/mouse)was used according to the report that intraperitoneal injection of 100 ng/rat recombinant BDNF can effectively induce a decrease in colonic reaction threshold [16]. From the 21st day, the mice in the BDNF-treated and BDNF-treated stressed groups were treated daily by intraperitoneal injection with 1 µg recombinant BDNF (GenWay Biotech, Inc., USA). The treatment was continued until the day when mice were killed. The mice in other groups were injected with vehicle (0.9% NaCl). After the open field test on the 30th day, mice in all groups received 5 IU pregnant mare serum gonadotropin (PMSG) intraperitoneally, followed with 10 IU human chorionic gonadotropin (hCG) 48 hours later.

The mice used for evaluation of BDNF expression were killed 6 hours after hCG injection. Animals were decapitated and trunk blood was collected, and plasma was stored at −80°C until the time of corticosterone assay. Left ovaries for western blotting were dipped into liquid nitrogen and stored at −80°C. Right ovaries and brains for immunohistochemistry were fixed in 4% paraformaldehyde at 4°C. The fixed ovaries were dehydrated and embedded in paraffin. Serial 3 mm sections were cut on a Leica microtome (Leica RM 2135). The fixed brains were frozen in OCT embedding medium (Sakura Finetek, Torrance, CA, USA ) after infiltration with 30% sucrose, Serial 20 µm frozen sections were cut on a Leica cryotome Cryostat (Leica CM 1900). The mice used for evaluation of oocyte developments were killed 14 hours after hCG injection, and their ovulated Cumulus–oocyte complexes (COCs) for in vitro parthenogenetic activation were collected from the fallopian tubes.

### Open Field Test

Open field activity, i.e. the initial activity of a animal placed in novel surroundings has long been taken as an indicator of its emotional and psychological state [17]. Protocols of open field test were previously approved [9]. The open field test was performed 12 h after ceasing the chronic stress procedure between the second and fifth hours of the dark phase. The apparatus consisted of a rectangular area of 81 × 81 cm surrounded by a 28 cm high wall. The area was divided into 16 squares of 20 × 20 cm by painted white lines. The field was lighted with a 40W bulb fixed 50 cm above the field. Light was focused on the center of the field with the periphery remaining dark. The mice were placed in one corner of the open field and its activity during the subsequent 5 min was assessed. Horizontal locomotion (number of times crossings of the white lines), frequency of rearing or leaning (sometimes termed vertical activity) and wall time (the time in the peripheral squares of the open field) were observed.

### Corticosterone Enzyme Immunoassay

Plasma corticosterone was measured using a competitive enzyme immunoassay (EIA) kit (NO. 500655, Cayman Chemical Co., USA), according to manufacturer’s instructions. In brief, the samples were washed and extracted with methylene chloride; placed in wells coated with rabbit antiserum with a competitive tracer; and concentration was assessed by using a spectrophotometer (ELX800, Biotek) measuring absorbance at 412 nm and comparing samples with known dilutions.

### Western Blotting

The ovaries were homogenized in ice-cold homogenization buffer (HB) containing 50 mM 3-(N-morpholino) propanesulfonic acid (pH 7.4), 100 mM KCl, 320 mM sucrose, 0.5 mM MgCl2, 0.2 mM dithiothreitol, 20 mM β-glycerophosphate, 20 mM sodium pyrophosphate, 50 mM NaF, 1 mM each of EDTA and EGTA, and protease inhibitor cocktail (11873580001, Roche, Mannheim, Germany). After the protein concentration was measured by the method of Lowry with bovine serum as standard [18], each sample was diluted to equal protein concentrations with HB. After adding 4×sodium dodecyl sulfate-polyacrylamide gel electrophoresis (SDS-PAGE) sample buffer into the sample, the sample was boiled in 100°C water for 10 min. Protein (50 µg) was loaded onto each lane, separated by 15% SDS-PAGE, and transferred onto a polyvinylidene difluoride membrane (Amersham Biosciences, UK). The membrane was blocked with 5% skimmed milk for 2 h, and then probed with rabbit poloclonal anti-BDNF antibody (1:500, ab72439, ABcam,USA ) or mouse monoclonal α-tubulin (1:1000 dilution, sc-23948, Santa cruz,USA) at 4°C overnight. Detection was performed using horseradish peroxidase(HRP) conjugated goat anti-mouse IgG (1:2000 dilution, P0260, Dako, A/S, Denmark) or HRP conjugated goat anti-rabbit IgG (1:2000 dilution, P0048, Dako, A/S, Denmark), and visualized by an ECL method using ECL Western Blotting Substrate (Promega). The bands on the X-ray film were scanned. BDNF bands were normalized relative to α-tubulin.

### Immunohistochemisty

For immunohistochemical detection of corticotropin-releasing hormone (CRH), brain sections were incubated in 0.3% H_2_O_2_ solution and blocked with 10% normal goat serum in 0.1% Triton X-100. Then the sections were incubated overnight with rabbit poloclonal anti-CRH antibody (1:1000, T-4037, Bachem Inc., Bubendorf, Switzerland) at 4°C. After washing, sections were incubated for 2 h with HRP conjugated horse anti-rabbit IgG (1:2000 dilution, P0048, Dako, A/S, Denmark) at room temperature, visualized with DAB/(NH_4_)_2_Ni(SO_4_)_2_, dehydrated in ethanol, and mounted in Entellan.

For immunohistochemical detection of BDNF, the sections were treated with microwaves (700 W) in 0.05 M citrate-buffered saline (pH 6.0) for 2 ×10 min for antigen retrieval. After incubating in 0.3% H_2_O_2_ solution and blocking with 10% normal goat serum in 0.1% Triton X-100, sections were incubated overnight with rabbit poloclonal anti-BDNF antibody (1:100, ab72439, ABcam,USA ) at 4°C. After washing, sections were incubated for 2 h with HRP conjugated horse anti-rabbit IgG (1:2000 dilution, P0048, Dako, A/S, Denmark) at room temperature, visualized with DAB, dehydrated in ethanol, and mounted in Entellan.

**Figure 1:**
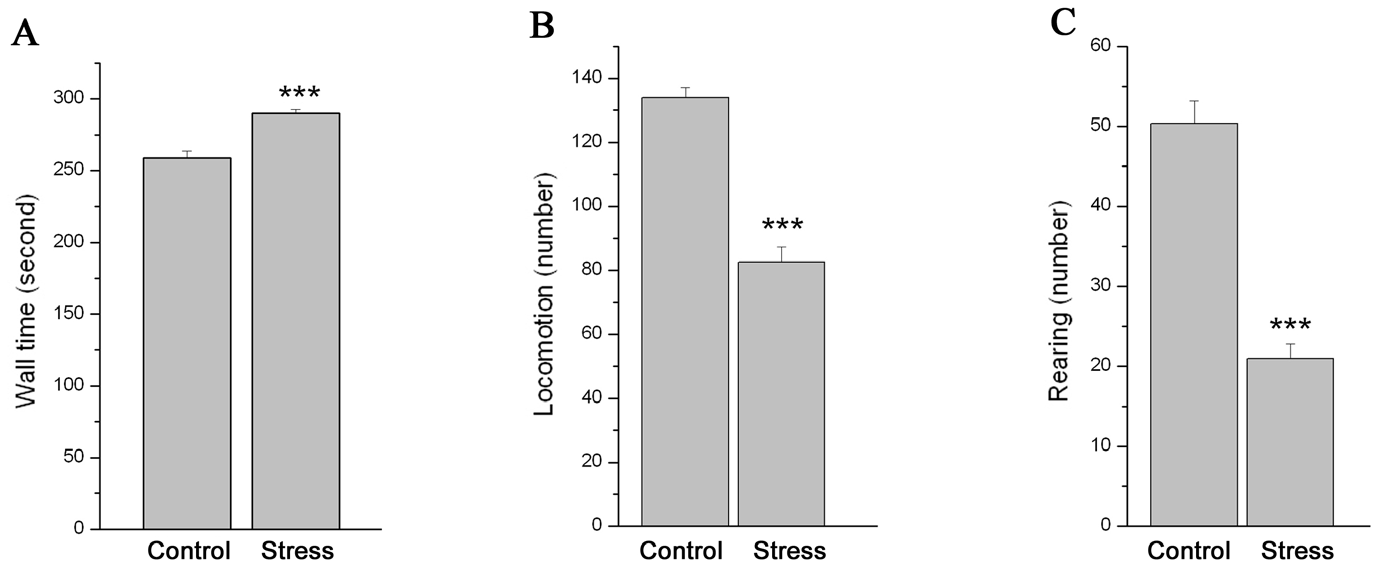
The effect of chronic stress on the behavior of mice in open field. Data (mean ± SEM) (n = 18) show wall time (A), the number of horizontal locomotion (B) and rearing (C) in 5 min in open field in control group and stressed group. *** P<0.001 vs. control group.

### Quantitation of Immunohistochemisty

An image analysis system was used, including MetaMorph image acquisition and processing software (Universal Imaging Corp.), a spot cooled color digital camera (Diagnostic Instruments, Inc.), a Nikon E800u fluorescence microscope (Nikon Corporation, Japan) equipped with a Prior scanning stage (Prior Scientific Instruments Ltd., England), and a HP computer.

To assess the total number of CRH neurons in the paraventricular nucleus (PVN) of the hypothalamus, serial sections were stained and all the blue staining CRH immunoreactive cells in PVN were counted.

To assess BDNF expressions, ovarian sections were obtained every 20th section. The expression of BDNF was quantified by measuring the average optical density (OD) in different stages of follicles. The follicles were classified into four stages according to the modified Oktay system [9]: ‘primordial follicle’  = an oocyte that was encapsulated by flattened pre-granulosa cells; ‘primary follicle’ = when at least one of the pre-granulosa cells has become columnar or cubic until they form a single layer of cubic granulosa cells; ‘secondary follicle’ = when the oocyte is encapsulated by two or more layers of granulosa cells without antrum formation; and ‘antral follicle’ = when the oocyte is encapsulated by more than two layers of granulosa cells and an antrum has formed. Total areas of follicles were analyzed, including areas of granulosa cells, the antrum and oocyte. The quantitative data are presented as folds change relative to control group.

**Figure 2:**
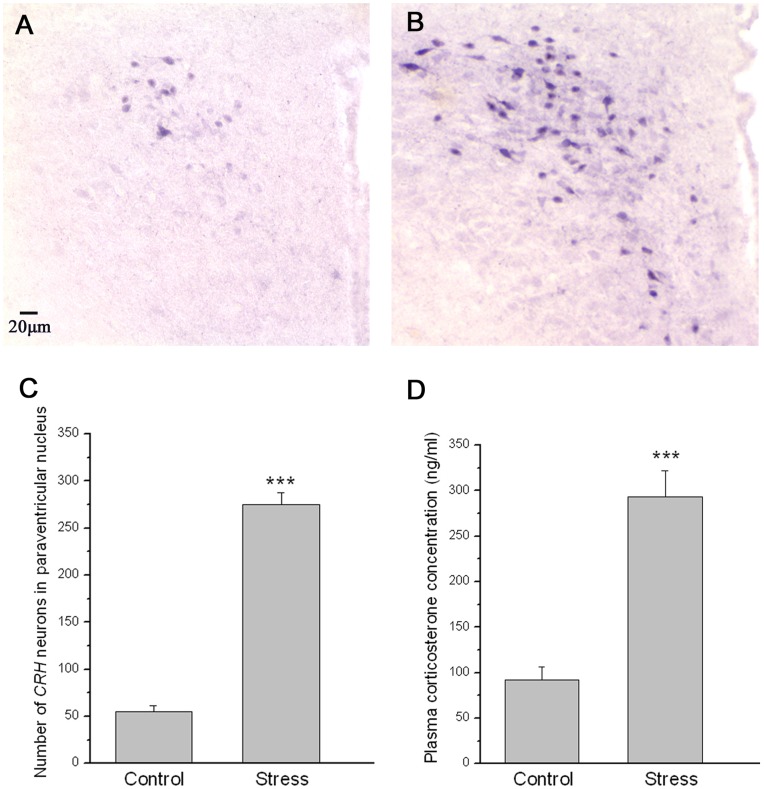
The effect of chronic stress on HPA axis activity of mice. Immunohistochemistry showed the CRH neurons in the paraventricular nucleus of the hypothalamus in control (Figure 2A) and stressed (Figure 2B) mice. Figure 3C showed the quantitative analysis of the total number of CRH neurons in PVN (mean ± SEM). Figure 3D showed the quantitative analysis of the plasma corticosterone concentration in control and stressed mice (mean ± SEM) (n = 9).

### Parthenogenetic Activation and Blastocyst Culture

To avoid the effects from sperm, our study used parthenogenesis rather than insemination to get embryos and blastocysts for assessing oocytes developmental potential. Further development to the blastocyst stage can be obtained when parthenogenetic embryos were developed. Blastocyst formation rate was a marker of oocytes developmental potential [19]. Before activation, cumulus-oocyte complexes were treated with 40 IU/ml of hyaluronidase (SAGE In Vitro Fertilization, Inc., USA) for removing cumulus cells. Cumulus-free oocytes were activated with 7% alcohol (Sigma-Aldrich, St. Louis, MO, USA) in Fertilization Medium (William A. COOK Australia Pty. Ltd, Australia) for 5 min at 37°C [20]. They were subsequently washed three times with Fertilization Medium (William A. COOK Australia Pty. Ltd, Australia), and then were cultured together in COOK Media under mineral oil in 35-mm Petri dishes (Falcon 1008; Becton & Dickinson, Lincoln Park, NJ) at 37°C in 6% CO2 incubator. Briefly, they were cultured in COOK Fertilization Medium overnight, then transferred into COOK Cleavage Medium on day 1 and transferred into COOK Blatocyst Medium on day 3. Mouse embryos will reach the blastocyst stage on day 4.

**Figure 3:**
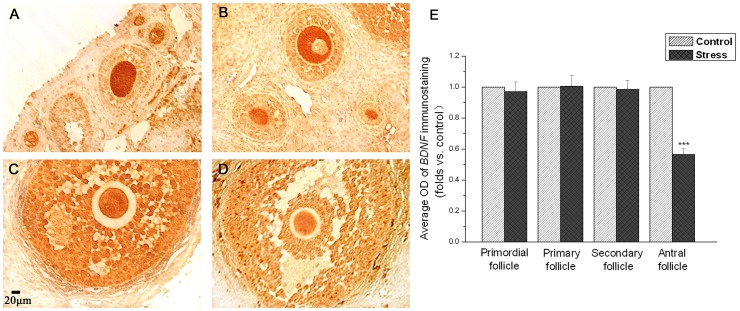
The effect of chronic stress on the ovarian BDNF detected by immunohistochemistry. Figure 3A and figure 3B show the ovarian BDNF immunoreactivity in early follicles in control (Figure 3A) and stressed (Figure 3B) mice. Figure 3C and figure 3D show the ovarian BDNF immunoreactivity in late follicles in control (Figure 3C) and stressed (Figure 3D) mice. Figure 3E shows the quantitative data (mean ± SEM) (n = 9) are shown as folds vs. control group. *** *P*<0.001 vs. control group.

**Figure 4:**
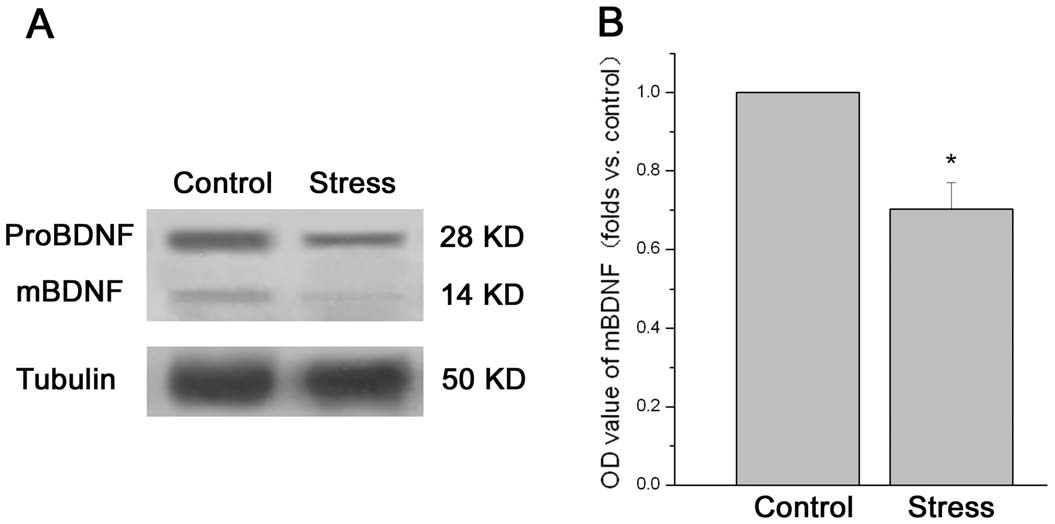
The effect of chronic stress on the ovarian BDNF detected by western blotting. Data (mean ± SEM) (n = 9) are shown as folds vs. control group. Figure 4A shows a representative western blot of ovarian BDNF. The predominant bands of 28 kD represent proBDNF, and the faint bands at 14 kD represent mature BDNF (mBDNF). Figure 4B shows the relative quantitative level of mBDNF protein. * *P*<0.05 vs. control group.

### Statistical Analyses

The results were shown as mean ± SEM (standard errors from the mean). The data were analyzed by one way or two-way ANOVA (stress × BDNF), followed by Tukey’s honestly significantly different (HSD) test as post hoc analysis for further examination of group differences. The rate of ocyte maturation and embryo cleavage were evaluated with Chi Square test. Significance was defined as *P*<0.05. All analyses were conducted by statistical software, SPSS 17.0 for Windows.

## Results

### 1. The Mouse Stressed Model is Validated by Open Field Test and HPA Axis Activity

The data of the wall time (Figure 1A), the number of horizontal locomotion (Figure 1B) and rearing (Figure 1C) from open field were shown in figure 1. Analysis showed that the wall time significantly increased, while the number of horizontal locomotion and rearing significantly decreased in stressed mice as compared to control mice (n = 18; *P*<0.001 for all).

**Table 1:** The effect of chronic stress and BDNF on the number of retrieved oocytes, oocyte maturation and embryo cleavage.

Group	Retrieved oocytes	Rate of MII oocytes	Rate of embryo cleavage
Control	31.89±2.04	99.30%	94.43%
Stressed group	17.11±1.49***	99.35%	93.51%
BDNF-treated group	31.56±2.02	99.30%	95.42%
BDNF-treated stressed group	24.89±1.13* **^#^**	99.55%	95.09%

The presented data of retrieved oocytes are the mean ± SE (n = 9). *P<0.05, *** P<0.001 vs. control group. #P<0.05 vs. stressed group.

The HPA axis activity was assessed by the number of CRH neurons in PVN of hypothalamus (Figure 2 A,B,C) and plasma corticosterone concentration(Figure 2D). Immunohistochemistry showed the number of CRH neurons in PVN significantly increased in stressed mice (Figure 2B) when compared with control mice (Figure 2A) (*P*<0.001). A quantitative analysis of the total number of CRH neurons in PVN was shown in figure 2C. The plasma corticosterone concentration was shown in figure 2D, which demonstrated that the plasma corticosterone concentration in stressed mice is significantly higher than that in control mice (*P*<0.001).

### 2. Ovarian BDNF Expression was Decreased after Chronic Unpredictable Stress

Immunohistochemistry (Figure 3A,B,C,D) showed abundant BDNF expression in ovary. There are regional differences in the level of BDNF protein in different developmental stages of follicles. BDNF immunoreactivity was distributed mainly in oocytes, but not granulose cells in primordial, primary and secondary follicles (Figure 3A and figure 3B). There are no differences in the expression intensity in primordial, primary and secondary follicles between control mice (Figure 3A) and stressed mice (Figure 3B). BDNF immunoreactivity was distributed in both oocytes and granulose cells in antral follicles (Figure 3C and figure 3D). The BDNF expression intensity in antral follicles in control mice (Figure 3C) looks much higher than that in stressed mice (Figure 3D). A quantitative analysis of BDNF expression in primordial, primary, secondary and antral follicles was shown in figure 3E. It appears that there are no differences in the average OD of BDNF immunoreactivity in primordial (*P* = 0.721), primary (*P* = 0.959) and secondary (*P* = 0.860) follicles between stressed mice and control mice. However, BDNF immunoreactivity significant decreased in antral follicles in stressed mice as compared to control mice (*P*<0.001). The average OD value of BDNF immunoreactivity in antral follicles in control mice was about twice more than that in stressed mice (Figure 3E).

Figure 4A showed a representative western blot of ovarian BDNF. The predominant bands of 28 kD represent proBDNF, and the faint bands at 14 kD represent mature, processed BDNF (mBDNF). The relative protein level of mBDNF in ovary was shown in figure 4B. Analysis showed that the protein levels of mBDNF in stressed mice were significantly decreased as compared to control mice (n = 9; P = 0.012).

### 3. Chronic Unpredictable Stress Decreased the Number of Retrieved Oocytes, while Treatment with BDNF Increased the Number of Retrieved Oocytes in Stressed Mice

The results presented in table 1 revealed the influence of chronic stress and BDNF upon the number of retrieved oocytes, oocyte maturation and early embryo cleavage. Two-way ANOVA (stress × BDNF treatment) showed a significant main effect of stress on the number of retrieved oocytes (F1, 32 = 39.096, *P*<0.001). The analysis also revealed a significant main effect of BDNF treatment on the number of retrieved oocytes (F1, 32 = 4.712, *P* = 0.037). There was a significant interaction between stress and BDNF treatment (F1, 32 = 5.593, *P* = 0.024).

Further analysis showed that the retrieved oocytes number in stressed mice significantly decreased as compared to control mice (*P*<0.001). There was a significant increase in the number of retrieved oocytes in the BDNF-treated stressed mice as compared to stressed mice (*P* = 0.015). Compared to control mice, there was still a significant decrease in the retrieved oocytes number in the BDNF-treated stressed mice (*P* = 0.033).

There are no significant differences in the oocyte maturation and early embryo cleavage between groups (*P*>0.05 for all comparisons).

**Figure 5:**
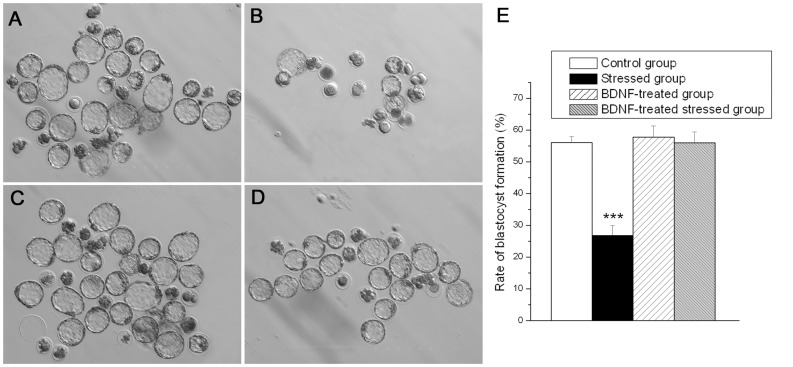
The effect of chronic stress and BDNF on the oocytes developmental potential. Figure 5 shows the representative oocytes formation in control (A), stressed (B), BDNF-treated (C) and BDNF-treated stressed (D) group. The presented data in figure 5E are average oocytes formation rate (mean ± SEM) (n = 9). *** *P*<0.001 vs. control group.

### 4. Chronic Unpredictable Stress Decreased the Oocytes Developmental Potential, while Treatment with BDNF Restored the Oocytes Developmental Potential in Stressed Mice

Blastocyst formation rates are associated with oocytes developmental potential. Representative images of blastocyst formations in control (A), stressed (B), BDNF-treated (C) and BDNF-treated stressed (D) group were shown in figure 5. A quantitative analysis of blastocyst formations rate was shown in figure 5E. The results presented in figure 5 revealed the influence of chronic stress and BDNF upon the oocytes developmental potential.

Two-way ANOVA (stress × BDNF treatment) showed a significant main effect of stress on the blastocyst formation rates (F1, 32 = 25.190, *P*<0.001). The analysis also revealed a significant main effect of BDNF treatment on the blastocyst formation rates (F1, 32 = 25.058, *P*<0.001). There was a significant interaction between stress and BDNF treatment (F1, 32 = 19.784, *P*<0.001).

Further analysis showed that the blastocyst formation rates in stressed mice significantly decreased as compared to control mice (*P*<0.001). There was a significant increase in the blastocyst formation rates in the BDNF-treated stressed mice as compared to stressed mice (*P*<0.001). There was no difference in the blastocyst formation rates between control mice and BDNF-treated stressed mice (*P* = 1.000).

## Discussion

The objective of this study was to evaluate the effects of chronic stress on ovarian BDNF expression and oocytes developmental potential. The major findings were that chronic unpredictable stress decreased the BDNF expression in antral follicles, but didn’t affect the BDNF expression in primary and secondary follicles. Chronic unpredictable stress also decreased the number of retrieved oocytes and subsequent oocytes developmental potential, which was rescued by exogenous BDNF treatment.

In the present studies, we induced psychosocial stress in mice using chronic unpredictable mild stress [14]. The initial activity of an animal placed in a novel surrounding (ie open field activity) has long been taken as an indicator of its psychological and emotional state [17]. For example, spending longer time in peripheral squares of the open filed indicates an anxiety-like activity, and reduced locomotion and exploratory activity represent a loss of interest in new stimulating situations, implying a deficit in motivation [17], [21]. The open field activity of the mice in our study implicated that the mice were in an anxious emotional and psychological state after chronic unpredictable stress. These data is consistent with our previous study [9], [15]. Consistent with the previous report [14], the animal model in our study also showed signs of increased activity in the HPA axis, including increased CRH neurons in PVN of hypothalamus and corticosterone hypersecretion from adrenal. The behavioral performance in open-field and the HPA axis hyperactivity of the mice in our study implicated that the stress model was established successfully.

BDNF, one of the neurotrophins, was originally described in the nervous system but has been shown to be expressed in a variety of nonneuronal tissues including endocrine tissues recently. The presence and secretion of BDNF from follicular cells in the human ovary were confirmed for the first time in 2002 [3]. Consistent with the previous report [6], our data showed a regional difference in the BDNF distribution in ovaries. The intensity of BDNF immunoreactivity in granulose cells was stronger in antral follicles than that in early follicles. A large number of studies had suggested that BDNF in the nervous system is a stress-responsive intercellular messenger that may be an important component of the stress response [11], [22], [23]. Furthermore, our data revealed for the first time that BDNF in ovary also is responsive to chronic stress.

Up to now the impaired ovarian function induced by chronic stress are mainly manifested as inhibition of gonadotropin release in anterior pituitary gland [24]–[26]. Little is known about the effect of interovarian regulators during chronic stress, although the developments of follicle and oocytes are regulated by both gonadotropin from pituitary gland and paracrine/autocrine regulators in ovary [27]. Many reports had demonstrated that BNDF is a paracrine/autocrine regulator that is required for follicular growth and oocyte development in the mammalian ovary [4]–[7], [28], [29]. Recently it has been proved that BDNF is involved in not only stress-related mood disorder [13], but also some human reproductive diseases, such as polycystic ovary syndrome [30] and infertility [31]. Our data showed for the first time that BDNF in ovary may be involved in the impaired oocytes developments induced by chronic stress.

Some researches showed that BDNF play a role in not only oocytes developmental potential, but also oocyte maturation and early embryo cleavage [4]–[7]. However, our data showed that chronic stress and the decreased BDNF expression induced by chronic stress only accounted for oocytes developmental potential, but didn’t affect oocyte maturation and embryo cleavage. This difference may be explained by using different technique and methods. Contrasted with the technique of in vitro maturation in the above researches [4]–[7], the methods of in vivo maturation in animal model of chronic stress and in vitro parthenogenetic activation were used in our study.

There was still a significant decrease in the retrieved oocytes number in BDNF-treated stressed mice as compared to control mice despite a significant increase when compared to stressed mice. These data implied that there may be some other mechanisms involved in the follicular maldevelopment besides BDNF during chronic stress. A study has shown that another ovarian regulator, GDF9 is involved in the follicular maldevelopment induced by chronic stress [9].

Infertile women are usually more depressed, anxious and may be hostile [32], [33]. It is likely that stress may have an adverse impact on the IVF outcome in spite of a new meta-analysis which contradicts this opinion [34].Our current studies indicate that supplement with intraovarian endocrine/paracrine regulators, such as BDNF, may be beneficial for oocytes developments when gonadotropin is used to hyperstimulate ovaries in women with chronic stress or mood disorder. More direct evidence from humans was expected.
